# Using weak signals to predict spontaneous breathing trial success: a machine learning approach

**DOI:** 10.1186/s40635-025-00724-0

**Published:** 2025-03-18

**Authors:** Romain Lombardi, Mathieu Jozwiak, Jean Dellamonica, Claude Pasquier

**Affiliations:** 1 Critical Care Unit, Pasteur 2 University Hospital, 30 Voie Romaine, 06000 Nice, France; 2 Critical Care Unit, Archet 1 University Hospital, 151 Rte de Saint-Antoine, 06200 Nice, France; 3 Université Côte d’Azur, UR2CA, Unité de Recherche Clinique Côte d’Azur, Nice, France; 4 I3S, CNRS, 2000 route des Lucioles, 06900 Sophia Antipolis, France

**Keywords:** Mechanical ventilation, Weaning, Spontaneous breathing trial, Machine learning, Biosignal, Weak signals, ICU, Critical care

## Abstract

**Background:**

Weaning from mechanical ventilation (MV) is a key phase in the management of intensive care unit (ICU) patient. According to the WEAN SAFE study, weaning from MV initiation is defined as the first attempt to separate a patient from the ventilator and the success is the absence of reintubation (or death) within 7 days of extubation. Mortality rates increase with the difficulty of weaning, reaching 38% for the most challenging cases. Predicting the success of weaning is difficult, due to the complexity of factors involved. The many biosignals that are measured in patients during ventilation may be considered “weak signals”, a concept rarely used in medicine. The aim of this research is to investigate the performance of machine learning (ML) models based on biosignals to predict spontaneous breathing trial success (SBT) using biosignals and to identify the most important variables.

**Methods:**

This retrospective study used data from two centers (Nice University Hospital, Archet and Pasteur) collected from 232 intensive care patients who underwent MV (149 successfully and 83 unsuccessfully) between January, 2020 and April, 2023. The study focuses on the development of ML algorithms to predict the success of the spontaneous breathing trial based on a combination of discrete variables and biosignals (time series) recorded during the 24 h prior to the SBT.

**Results:**

For the models tested, the best results were obtained with Support Vector Classifier model: AUC-PR 0.963 (0.936–0.970, *p* = 0.001), AUROC 0.922 (0.871–0.940, *p* < 0.001).

**Conclusions:**

We found that ML models are effective in predicting the success of SBT based on biosignals. Predicting weaning from mechanical ventilation thus appears to be a promising area for the application of AI, through the development of multidimensional models to analyze weak signals.

**Supplementary Information:**

The online version contains supplementary material available at 10.1186/s40635-025-00724-0.

## Take Home Message

- The concept of “weak signals” is underused in medicine, because interpreting them is complex. The use of machine learning models to identify and interpret weak signals produces promising results
- Predicting the success of weaning is crucial for the management of patients on mechanical ventilation. This study suggests the application of multidimensional machine learning models to routinely collected biosignals could help predict spontaneous breathing trial success.

## Background and significance

Endotracheal intubation is one of the most common resuscitation procedures and can be necessary for up to 90% of patients admitted to an intensive care unit (ICU), depending on the country [1]. This procedure has been shown to cause several complications, including: severe hypoxemia, severe arterial hypotension and hypoxic cardiac arrest [2, 3]. The weaning period is a key stage in the management of patients on mechanical ventilation (MV) and can take up half of the hospital stay. Weaning is defined as the first attempt to remove a patient from the ventilator, and success is the absence of reintubation or death within 7 days after extubation [4–6]. Spontaneous breathing trial (SBT) is commonly used to assess patient’s readiness to be weaned. Up to 35% of patients subsequently experience extubation failure [7]. Regardless of the risks of reintubation, the mortality rate increases dramatically with the difficulty of weaning and reaching 38% in patients with most difficult weaning [8, 9]. Furthermore, the longer weaning is delayed, the higher is the length of hospital stays and the higher is the risk of failure [8]. Therefore, more reliable predictions of weaning success would not only assist clinicians and improve patient outcomes, but could also potentially have an economic impact by reducing hospital stays.

Artificial intelligence (AI) has already found several applications in various areas of medicine, particularly in the field of critical care, such as the management of fluid administration and vasopressors in patients with septic shock, prediction of sepsis or management of acute kidney injury [10–16]. However, data on the use of AI, and in particular machine learning (ML), in the MV weaning process are still scarce [14, 17, 18]. ML is a branch of AI characterized by models that learn based on data [19, 20]. The goal of ML is to discover recurring patterns in data sets, such as numbers, words or images in order to make predictions about new data [21]. ML algorithms can be divided into two categories, supervised and unsupervised. Supervised algorithms, further divided into classification and regression algorithms, are based on learning with labeled data [20, 22]. Unsupervised algorithms, divided into clustering and dimensional reduction algorithms, do not require data labeling [22]. Several literature reviews have examined the potential impact of ML in everyday medical practice and in biology [23–25].

A key area in which ML may advance medical practice is in identifying and interpreting weak signals. Weak signals, first defined by Ansoff in the late twentieth century, are signals that appear to be incomplete, unstructured and unprocessed [26]. They are early, low-intensity pieces of information that indicate an emerging trend. If detected and interpreted accurately, they allow future events to be anticipated and thereby facilitate an appropriate response. In general, their sources and natures can vary from environmental to biological [27]. In the context of MV weaning, biological data, such as respiration and heart rate, may be weak signals that could be used to predict patient outcomes if processed appropriately. They are not widely used in this way because they are difficult to interpret, but they are interesting and original sources of data [28–32]. Few studies have used them to predict MV weaning outcomes [33, 34].

The aim of this research was to investigate the performance of ML models based on biosignals in predicting the success of SBT and identifying the most important variables.

## Objectives

We aim to develop different ML models to predict SBT success or failure based on variables that were routinely collected during ICU patient care. Then, we assess the performance of the models and identify which variables are most important in predicting SBT success.

## Materials and methods

### Data collection and study sample

This retrospective study used clinical, biological and biosignal data collected from patients who underwent MV in ICU at Nice University Hospitals l’Archet and Pasteur 2, tertiary teaching hospitals in the South of France, from January 2020 to April 2023. Data prior to January 2020 were excluded due to high levels of missing data. All the data (clinical, biological and biosignal) were obtained directly from electronic medical records and did not require any additional measurements. Patients prior to 2020 were excluded because the data quality was insufficient.

We screened all patients admitted to the ICU who underwent MV. Patients were included if they were over 18 years of age and underwent at least one SBT. The SBT could be performed in the following ways: T-piece test, or with a pressure support ventilation, with a positive end expiratory pressure (PEEP) of 4 (PEEP4) or with PEEP of 0 (ZEEP) [5, 35]. Patients were excluded if: no electronic report of the result was found, the patient was transferred to another department before the SBT was performed, the withdrawal test was not clearly explained in the medical record, or self-extubation or death occurred before the SBT. If more than one SBT was performed, only the first was considered in this study.

Finally, we included a total of 232 patients in this study.

This study was approved by the French Intensive Care Ethic Committee (CE 23-017) and was registered on ClinicalTrials.gov (NCT05886803).

### Outcomes

The primary outcome of this study was the performance of different ML algorithms in predicting the success of the SBT. The criteria for SBT failure were agitation, altered mental status, respiratory rate > 35/min, signs of respiratory distress, hemoglobin oxygen saturation measured by pulse oximetry < 90%, or an increase of heart rate or blood pressure > 20% from baseline at the end of SBT [36].

The secondary outcome was to determine which features were important in the algorithms’ output.

### Predictors

We included both discrete and continuous variables (biosignals). The discrete variables were: demographics (sex, age, inclusion center), comorbidities, severity scores at admission (Simplified Acute Physiology Score II or SAPSII, Apache2, Sepsis-related Organ Failure Assessment or SOFA), main reason for admission, main reason for intubation, body mass index (BMI) at time of SBT, weight gain since ICU admission, weaning test type (ZEEP, PEEP4, T-tube), non-invasive ventilation prior to intubation, ventilation characteristics (total number of days of invasive MV, time between intubation and first separation attempt, total number of days in volume-controlled ventilation (VCV) mode, total number of ventral decubitus, use of inhaled nitric oxide), use of drugs during SBT (purpose and dose), use of extra-renal replacement (type and duration), presence of ventilator-associated pneumonia (VAP) before SBT and biology at the time of SBT. The continuous variables measured as time series were heart rate, systolic blood pressure, diastolic blood pressure, mean arterial blood pressure, cumulative and hourly urinary output, glycemia, clinical pulmonary infection score (CPIS), temperature, Richmond Agitation Sedation Scale (RASS) score, respiratory rate, SpO2 and ventilatory parameters (FiO2, PEEP, minute-volume and tidal volume). Most parameters were measured in intervals of one minute. We arbitrarily chose to include data from 24 h prior to the SBT up until the start of the SBT. No additional measurements were required.

### Data processing

We dealt with missing values through multiple imputation using the K-nearest neighbors (KNN) method [37, 38]. To overcome the problem of the unbalanced dataset the synthetic minority oversampling technique (SMOTE) was used (based on the generation of virtual individuals to increase the representation of the minority class) [39–41].

To input the time series data into the ML models, we used the feature extraction based on scalable hypothesis tests (FRESH) method. Given the large number of variables (and therefore dimensions) being considered, we applied two types of dimensional reduction to reduce the training time of the models. A variable is considered relevant if it is not independent of the target to be predicted (based on a statistical test appropriate for that variable and a *p*-value < 0.05). We then took all the relevant features with *p*-values < 0.05 and referred to this as the “light dimensional reduction”. We tested another dimensional reduction, limiting the data to the first 20 relevant variables (smallest p-values, including both continuous and discrete variables), and refer to this as the “heavy dimensional reduction”. See Supplementary Methods for more details.

**Fig. 1:**
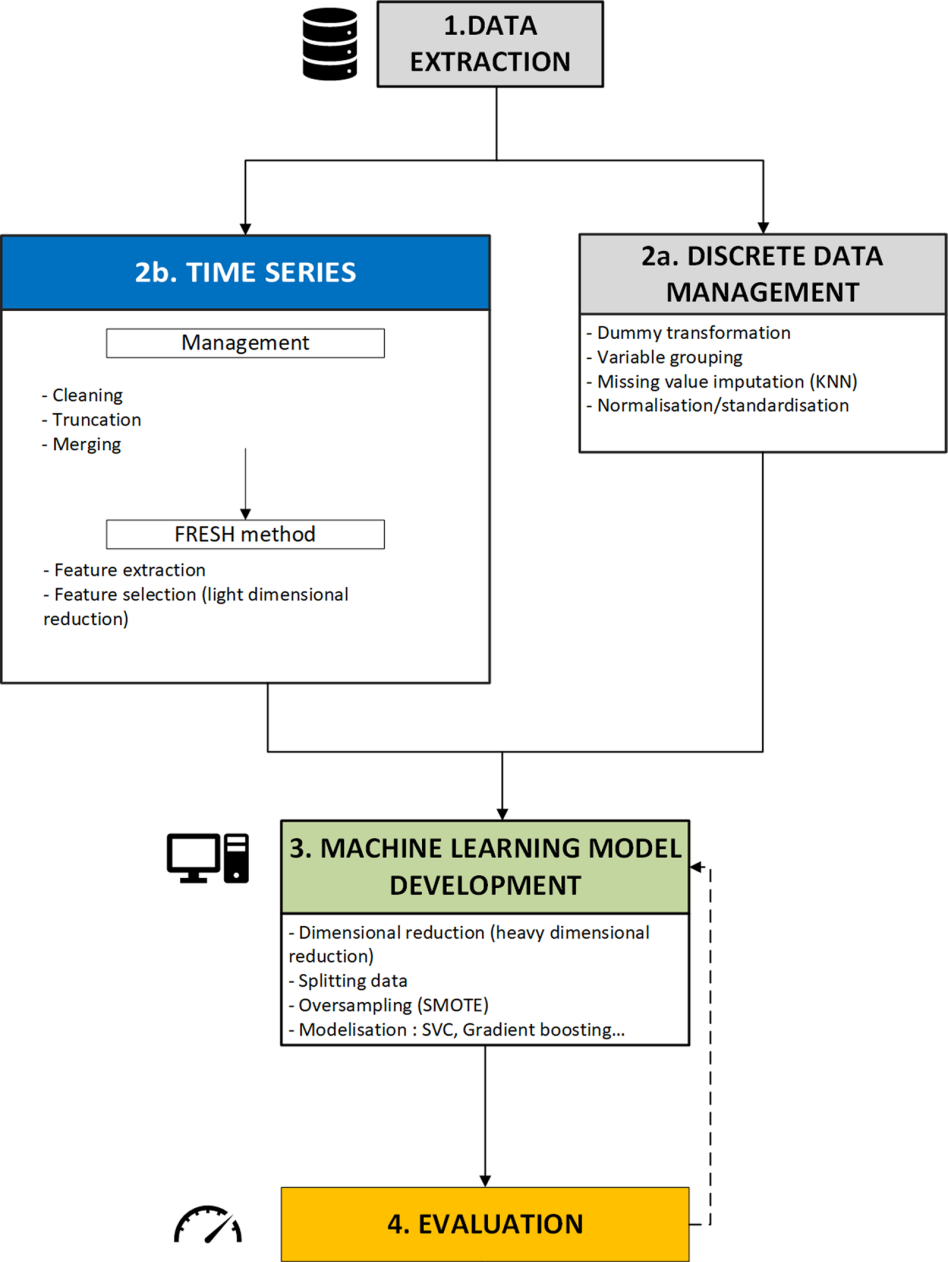
Conceptual workflow of the data preprocessing. Time series are biosignals. The dotted arrow indicates a feedback loop. *FRESH* FeatuRe Extraction based on Scalable Hypothesis tests, *KNN* K-nearest neighbors, *SMOTE* synthetic minority oversampling technique, *SVC* Support Vector Classifier

A conceptual diagram of the data processing is shown in Fig. 1.

### Training of machine learning models and statistical analysis

We used several ML models: Logistic Regression (LR), Random Forest Classifier (RFC), Support Vector Classifier (SVC), K-nearest neighbors (KNN), Gradient Boosting Machine models (eXtremely Gradient Boosting or XGBoost and Light Gradient Boosted Machine or LGBM), and a stacking classifier (ensemble model, combining Random Forest Classifier and Support Vector Classifier). The hyperparameters were tuned using cross-validation and grid search optimization. For training, we split the development cohort into 80% training and 20% test parts. A stratified split was also performed (maintaining the same success/failure ratio as in the overall dataset).

For the descriptive statistics of the population, continuous variables were expressed as median [interquartile range] or median ± standard deviation depending on the distribution, and categorical variables were expressed as number (percentage). We used a Shapiro–Wilk test to determine the normality of the continuous variables. We used either the *χ*^2^ test or Fisher’s exact test to compare categorical variables, and the Mann–Whitney *U* test to compare continuous variables. A *p*-value < 0.05 was considered to indicate a significant difference.

The following performance metrics were used to assess the ML models: area under the receiver operating curve (AUROC), area under the precision-recall curve (AUCPR), F1-score, sensitivity, specificity, positive predictive value (precision), negative predictive value and accuracy. We defined the minimum significance level for AUROC and AUCPR as 0.8. The confidence intervals were obtained with a bootstrap method (*n*-repetitions = 1000) and the AUCs were tested against random chance using a permutation method (*n*-repetitions = 1000).

The models were further explored using the Shapley additive explanations (SHAP) value, to determine the importance of different features (variables) in the model output [42].

To evaluate the influence of sample size, we tested the variation in AUROC and AUCPR values with different numbers of observations (25, 50, 75, 100, 125, 150, 175, 200 and total cohort).

### Development environment

All the development work was conducted using Python version 3.9.16, along with the following libraries and their respective versions: NumPy 1.23.5, pandas 1.5.3, matplotlib 3.7.1, seaborn 3.7.1, missingno 0.5.2, imblearn 0.10.1, joblib 1.1.1, tableone 0.7.12, TSfresh 0.20.0, Sklearn version 1.1.3, LightGBM 2.2.3, XGBoost 1.5.0, TensorFlow 2.12.0, SHAP 0.41.0.

## Results

### Patient characteristics

**Fig. 2:**
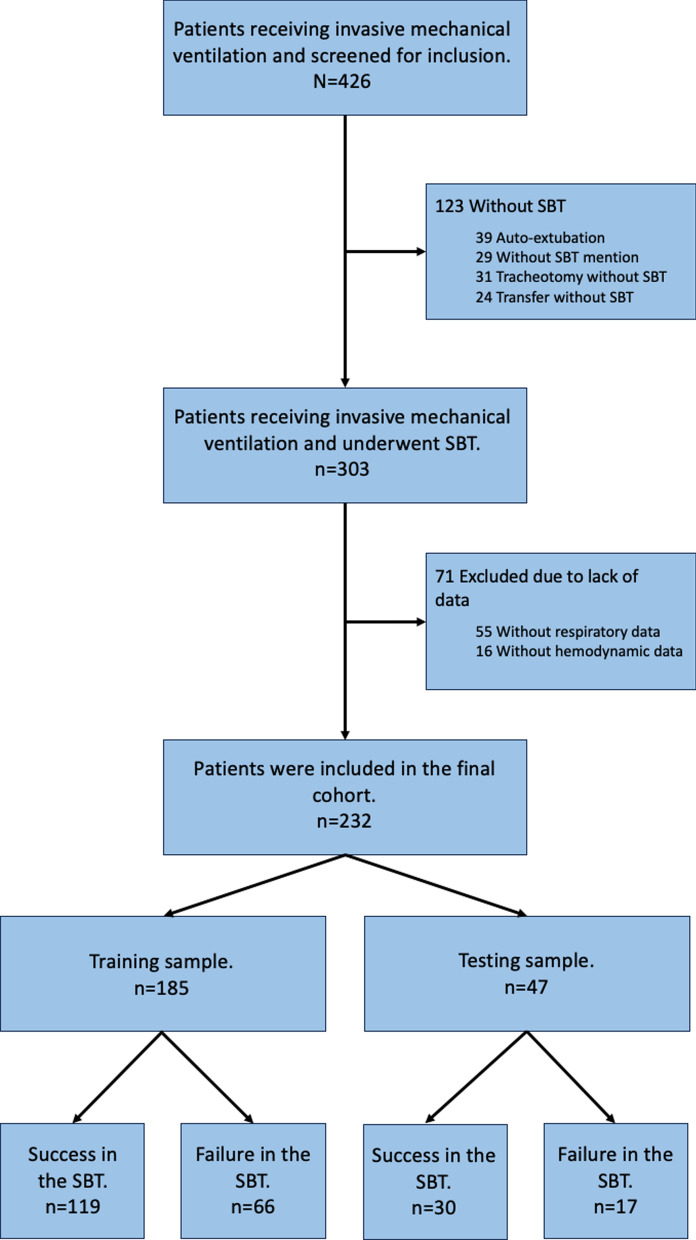
Study flowchart. *SBT* spontaneous breathing trial

Overall, 232 patients were included in the development cohort used to assess the ML models: 149 patients (64.2%) succeeded in the SBT and 83 (35.8%) failed, and a further 71 patients were excluded due to a lack of data (55 without respiratory data and 16 without hemodynamic data); see Fig. 2*.*

There was no significant difference between the two groups in terms of severity at admission (SAPSII: 54.2 vs. 54.7, *p* = 0.843, SOFA score: 8.5 vs. 9, *p* = 0.675). Patients in the success group were younger (63 years vs. 66 years, *p* = 0.032) and were more likely to be admitted for neurological reasons (29.5% vs. 13.3%, *p* = 0.008); see Table 1. Those who failed SBT tended to include a higher percentage of patients intubated mainly for respiratory reasons (74.7% vs. 54.4%, *p* = 0.004) and a lower percentage of neurological intubations (13.3% vs. 34.9%, *p* = 0.001). There were also more PEEP-ZEEP tests in the success group (62.4% vs. 42.2%, *p* = 0.003). There were more instances of VAP (71.1% vs. 19.5%, *p* < 0.001), longer hospital stays (13.9 days vs. 8.6 days, *p* < 0.001) and longer durations of volume-controlled ventilation (3 days vs. 2 days, *p* < 0.001) in the failure group compared with the success group. The delay from intubation to the first SBT was shorter for patients who passed their SBT (4 days vs. 6 days, *p* = 0.002). Patient characteristics and outcomes are summarized in Table 1.

**Table 1:** Patient characteristics and outcomes

Characteristics	SBT failure (*n* = 83)	SBT success (*n* = 149)	*p*-value
Characteristics of the patients at admission			
Age, median [Q1,Q3], (years)	66.0 [59.5,74.0]	63.0 [52.0,72.0]	**0.032**
Sex			
Female, *n* (%)	32 (38.6)	59 (39.6)	0.987
Male, *n* (%)	51 (61.4)	90 (60.4)	
SAPSII, mean (SD)	54.2 (18.6)	54.7 (18.1)	0.843
Apache2, median [Q1,Q3]	22.0 [16.0,28.5]	24.0 [18.0,30.0]	0.454
SOFA score, median [Q1,Q3]	8.5 [6.0,11.8]	9.0 [6.0,11.0]	0.675
BMI, median [Q1,Q3]	25.8 [22.8,29.3]	26.4 [22.9,29.8]	0.358
Comorbidities			
Immunosuppression, *n* (%)	14 (16.9)	30 (20.1)	0.664
Neurodegenerative disease, *n* (%)	7 (8.4)	14 (9.4)	0.995
Chronic hepatic disease, *n* (%)	12 (14.5)	22 (14.8)	1.000
Chronic kidney disease, *n* (%)	8 (9.6)	5 (3.4)	0.071
COPD, *n* (%)	19 (22.9)	23 (15.4)	0.217
Obstructive sleep apnea, *n* (%)	7 (8.4)	7 (4.7)	0.391
Arterial hypertension, *n* (%)	30 (36.1)	53 (35.6)	1.000
Diabetes mellitus, *n* (%)	20 (24.1)	34 (22.8)	0.953
Ischemic cardiopathy, *n* (%)	13 (15.7)	24 (16.1)	1.000
Dilated cardiopathy, *n* (%)	4 (4.8)		**0.016**
Hypertrophic cardiopathy, *n* (%)	2 (2.4)	3 (2.0)	1.000
Obstructive cardiopathy, *n* (%)	1(0.4)	1 (0.7)	1.000
Atrial fibrillation, *n* (%)	8 (9.6)	19 (12.8)	0.620
Valvulopathy, *n* (%)	4(1.7)	4 (2.7)	0.300
Main reason for ICU admission*			
Respiratory admission, *n* (%)	46 (55.4)	68 (45.6)	0.196
COVID19 admission, *n* (%)	24 (28.9)	44 (29.5)	1.000
Neurologic admission, *n* (%)	11 (13.3)	44 (29.5)	**0.008**
Cardiac Arrest admission, *n* (%)	6 (7.2)	12 (8.1)	1.000
Surgical admission, *n* (%)	1 (1.2)	1 (0.7)	1.000
Multivisceral failure admission, *n* (%)	5 (6.0)	6 (4.0)	0.529
Shock admission, *n* (%)	12 (14.5)	12 (8.1)	0.190
Main reason for intubation*			
Respiratory, *n* (%)	62 (74.7)	81 (54.4)	**0.004**
Neurological, *n* (%)	11 (13.3)	52 (34.9)	**0.001**
Surgical, *n* (%)	5 (6.0)	4 (2.7)	0.288
Cardiac arrest, *n* (%)	6 (7.2)	13 (8.7)	0.882
Characteristics of the SBT			
PEEP-ZEEP, *n* (%)	35 (42.2)	93 (62.4)	**0.003**
With PEEP4, *n* (%)	33 (39.7)	49 (32.9)	0.365
T-tube, *n* (%)	15 (18.1)	7 (4.7)	**0.002**
Biology at the time of the SBT			
pH, median [Q1,Q3]	7.5 [7.4,7.5]	7.5 [7.4,7.5]	0.337
PaCO2, median [Q1,Q3], mmHg	37.3 [34.0,41.8]	37.7 [34.6,41.0]	0.894
PaO2, median [Q1,Q3], mmHg	76.2 [68.3,87.0]	78.5 [71.5,89.4]	0.184
Bicarbonate, median [Q1,Q3], mmol/l	27.3 [25.0,30.2]	26.6 [23.5,29.3]	0.082
Arterial lactate, median [Q1,Q3], mmol/l	1.1 [0.7,1.4]	0.9 [0.7,1.4]	0.513
Albumin, median [Q1,Q3], g/l	23.5 [19.8,27.2]	25.1 [21.7,29.3]	**0.047**
Protide, median [Q1,Q3], g/l	60.0 [56.5,64.0]	57.0 [54.0,62.0]	**0.004**
Creatinine, median [Q1,Q3], µmol/l	72.0 [48.0,111.0]	67.0 [52.0,99.0]	0.880
Urea, median [Q1,Q3], mmol/l	10.5 [6.5,14.4]	8.7 [6.0,13.1]	0.180
Kaliemia, median [Q1,Q3], mmol/l	3.9 [3.6,4.1]	3.9 [3.5,4.1]	0.610
Natremia, median [Q1,Q3], mmol/l	140.0 [137.0,143.0]	140.0 [138.0,143.0]	0.529
Hemoglobin, median [Q1,Q3], g/dl	9.8 [8.7,11.6]	10.4 [8.8,12.0]	0.333
Hematocrit, median [Q1,Q3], l/l	0.3 [0.3,0.4]	0.3 [0.3,0.4]	0.383
Thrombocytes, median [Q1,Q3], ×10^9^/l	259.0 [180.0,372.0]	226.0 [152.0,322.0]	**0.024**
CRP, median [Q1,Q3], mg/l	51.2 [17.9,108.4]	39.4 [8.0,102.2]	0.183
PCT, median [Q1,Q3], ng/ml	0.3 [0.2,1.1]	0.2 [0.1,1.8]	0.242
Fibrinogen, median [Q1,Q3], g/l	5.8 [4.0,7.1]	3.3 [2.5,5.2]	**< 0.001**
Leukocytes, median [Q1,Q3], ×10^9^/l	11.8 [8.7,14.9]	11.6 [8.9,15.6]	0.917
Lymphocytes, median [Q1,Q3], ×10^9^/l	1.1 [0.6,1.6]	1.2 [0.7,1.6]	0.435
Outcomes			
VAP, *n* (%)	59 (71.1)	29 (19.5)	**< 0.001**
LOS, median [Q1,Q3], day	13.9 [7.9,24.3]	8.6 [5.4,12.7]	**< 0.001**
Deceased status, *n* (%)	7 (8.4)	6 (4.0)	0.232
Extubation failure, *n* (%)	9 (10.8)	18 (12.1)	0.946
Total no. of days of invasive MV, median [Q1,Q3], day	10.1 [5.8,18.9]	4.8 [2.6,9.0]	**< 0.001**
Delay from intubation to first SBT, median [Q1,Q3], day	6.0 [3.0,13.0]	4.0 [2.0,7.0]	**0.002**
Total no. of days of VCV mode, median [Q1,Q3], day	3.0 [2.0,7.0]	2.0 [1.0,4.0]	**< 0.001**

*BMI* body mass index, *COPD* chronic obstructive pulmonary disease, *CRP* C-reactive protein, *LOS* length of stay, *MV* mechanical ventilation, *PCT* procalcitonin, *PEEP* positive end expiratory pressure, *SAPSII* Simplified Acute Physiology Score, *SBT* spontaneous breathing trial, *SOFA* Sepsis-Related Organ Failure Assessment, *VAP* ventilator-associated pneumonia, *VCV* volume-controlled ventilation, *ZEEP* zero PEEP

Bold: *p*-value < 0.05

^*^Because multiple reasons are possible the total number exceeds the number of patients

### Accuracy and predictive power of the models

**Fig. 3:**
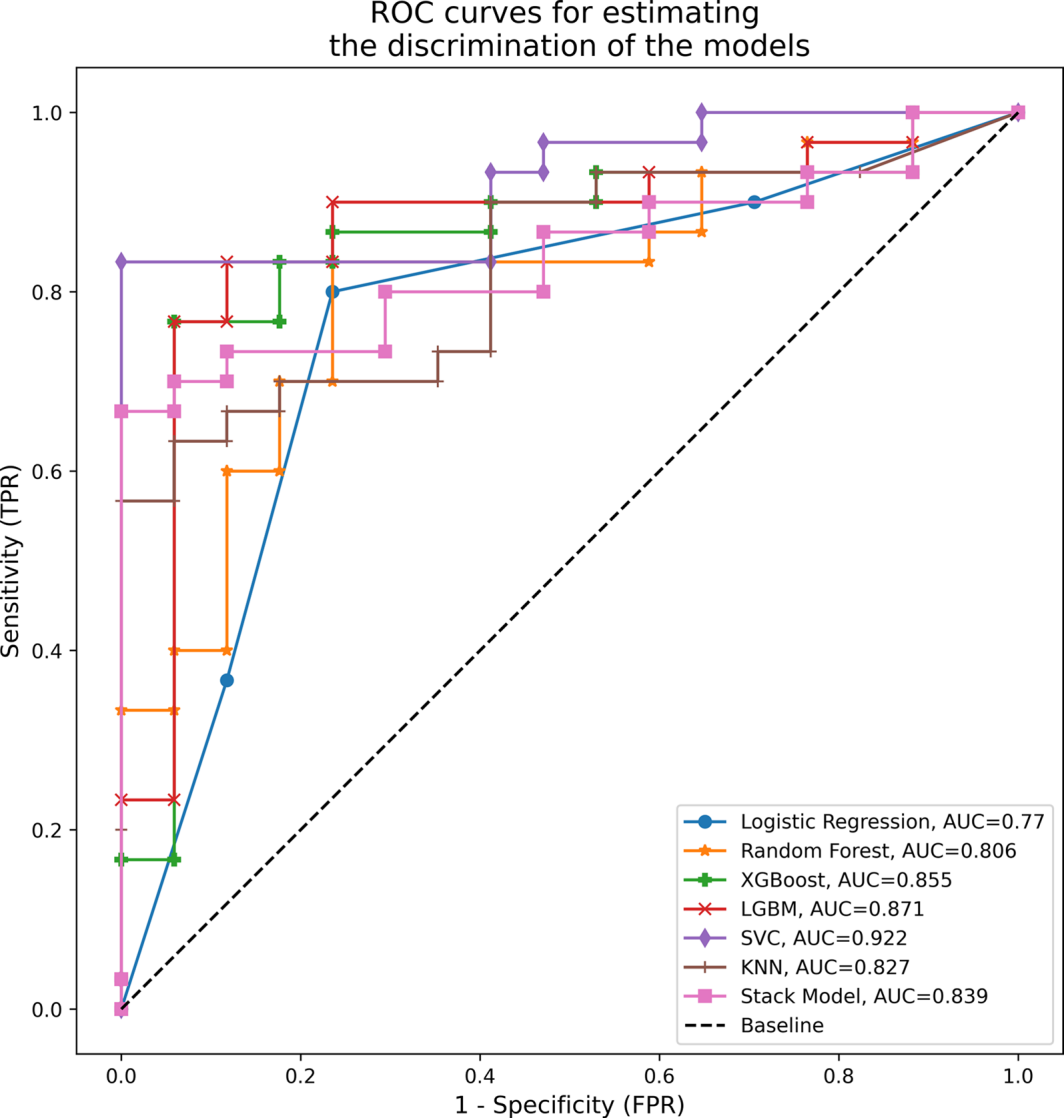
ROC AUC curves used to assess the models. We used multiple imputation, light dimensional reduction (238 features) and SMOTE on the test dataset. The Stack model contains a combination of Support Vector and Random Forest Classifier. *KNN* K-nearest neighbors, *LGBM* light gradient boosting machine, *SMOTE* synthetic minority oversampling technique, *SVC* support vector classifier, *XGBoost* extreme gradient boosting

**Fig. 4:**
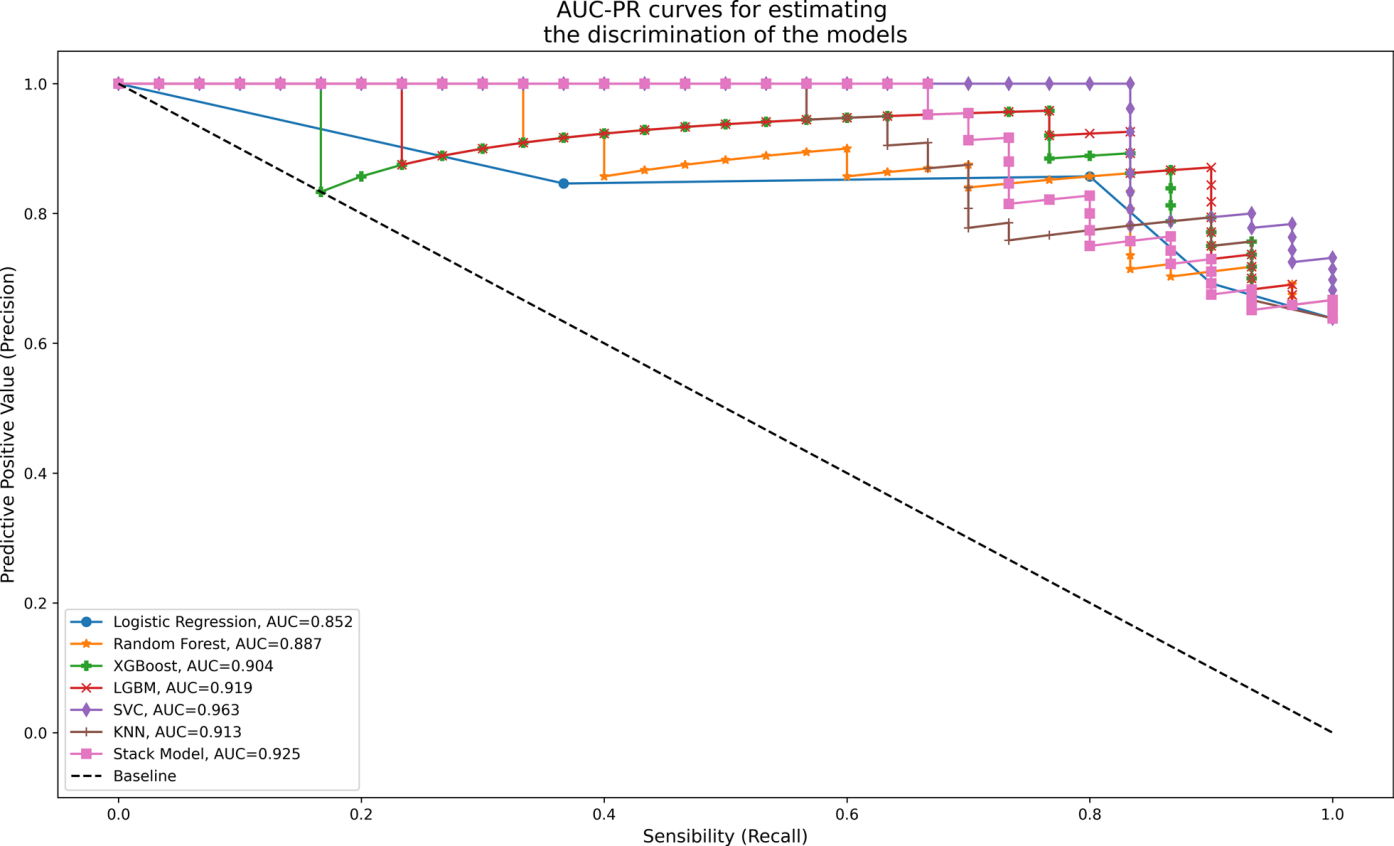
AUC precision-recall curves used to assess the models. We used multiple imputation, light dimensional reduction (238 features) and SMOTE on the test dataset. The Stack model contains a combination of Support Vector and Random Forest Classifier. *AUCPR* area under curve precision-recall, *KNN* K-nearest neighbors, *LGBM* light gradient boosting machine, *SMOTE* synthetic minority oversampling technique, *SVC* support vector classifier, *XGBoost* extreme gradient boosting

The results of the AUROC curves and AUCPR obtained for the different algorithms after applying the different pre-processing methods (imputation, SMOTE and light dimensional reduction) are shown in Figs. 3 and 4 and Table 2. These results represent the performance of the ML models on the test dataset after training on the training dataset. In terms of AUROC, the best predictions were obtained with the SVC model: 0.922 (0.871–0.940, *p* < 0.001), and followed by the LGBM: 0.871 (0.812–0.922, *p* < 0.001). The worst predictions were obtained with the Logistic Regression model with an AUROC of 0.77 (0.756–0.834, *p* < 0.001).

**Table 2:** Results of the different machine learning models

	F1-score	AUROC		AUCPR		Sp	Se	PNV	PPV
			*p*-value		*p*-value				
Light dimensional reduction (238 features) + SMOTE									
Logistic Regression	0.828	0.77	< *0.001*	0.852	*0.012*	0.765	0.8	0.684	0.857
95%CI		(0.756–0.834)		(0.821–0.904)					
Random Forest	0.82	0.806	< *0.001*	0.887	*0.001*	0.647	0.833	0.688	0.806
95%CI		(0.747–0.9)		(0.84–0.945)					
XGBoost	0.836	0.855	< *0.001*	0.904	*0.004*	0.882	0.767	0.682	0.920
95%CI		(0.804–0.912)		(0.871–0.960)					
LGBM	0.847	0.871	< *0.001*	0.919	*0.002*	0.765	0.833	0.722	0.862
95%CI		(0.812–0.922)		(0.880–0.963)					
SVC	0.806	**0.922**	< *0.001*	**0.963**	*0.001*	0.588	0.833	0.667	0.781
95%CI		(0.871–0.940)		(0.936–0.970)					
KNN	0.75	0.827	< *0.001*	0.913	*0.001*	0.706	0.7	0.571	0.808
95%CI		(0.702–0.853)		(0.815–0.925)					
Stack model*	0.820	0.839	< *0.001*	0.925	*0.001*	0.647	0.833	0.688	0.806
95%CI		(0.826–0.937)		(0.913–0.968)					
Heavy dimensional reduction (20 features) + SMOTE									
Logistic Regression	0.828	0.782	< *0.001*	0.892	*0.003*	0.765	0.8	0.684	0.857
95%CI		(0.761–0.845)		(0.830–0.912)					
Random Forest	0.781	0.728	< *0.001*	0.829	*0.017*	0.529	0.867	0.692	0.765
95%CI		(0.646–0.841)		(0.742–0.916)					
XGBoost	0.787	0.773	*0.001*	0.862	*0.005*	0.588	0.8	0.625	0.774
95%CI		(0.704–0.876)		(0.783–0.936)					
LGBM	0.767	0.714	*0.007*	0.813	*0.022*	0.588	0.767	0.588	0.767
95%CI		(0.641–0.812)		(0.716–0.897)					
SVC	0.844	0.796	< *0.001*	0.868	*0.007*	0.588	0.9	0.769	0.794
95%CI		(0.678–0.849)		(0.720–0.923)					
KNN	0.807	0.849	< *0.001*	0.916	*0.001*	0.765	0.767	0.65	0.852
95%CI		(0.733–0.886)		(0.835–0.936)					
Stack model*	0.852	0.825	< *0.001*	0.885	*0.003*	0.706	0.867	0.750	0.839
95%CI		(0.706–0.857)		(0.790–0.930)					

We used light dimensional reduction (238 features) and heavy dimensional reduction (20 features) on the test dataset

*AUCPR* area under curve precision-recall, *AUROC* area under the receiver operating curve, *KNN* K-nearest neighbors, *LGBM* light gradient boosting machine, *NBC* Naïve Bayes Classifier, *PNV* predictive negative value, *PPV* predictive positive value, *SMOTE* synthetic minority oversampling technique, *SVC* support vector classifier, *XGBoost* extreme gradient boosting

Bold: indicates the highest value in each column

^*^The Stack model is a combination of Support Vector and Random Forest Classifier

As shown in Fig. 4 and Table 2, the best AUCPR was also obtained by the SVC model: 0.963 (0.936–0.970, *p* = 0.001). The combination of RFC and SVC model in the Stack Model produced worse results than the SVC model alone (AUCPR: 0.929, 95% CI 0.912–0.970, *p* = 0.001).

The models performed worse when heavy dimensional reduction (20 features) was used; see Table 2*.* In this case, the KNN model obtained the highest AUCPR (KNN: 0.916, 95% CI 0.835–0.936, *p* = 0.001) and AUROC (KNN: 0.849, 95% CI 0.733–0.886, *p* < 0.001).

The calculation of SHAP values allowed us to determine the relative importance of different features in the models. In Supplementary Fig. 1, representing the 5 most important features, we can see that the presence of VAP before the SBT, fibrinogenemia at the time of SBT and weight gain since admission are decisive variables. The patients who failed the SBT were more likely to have VAP, and to have gained weight. The patients whose SBT was successful had lower fibrinogen levels than those the other group.

### Effect of sample size

We evaluated the importance of sample size variation in the performance, in terms of AUROC and AUCPR; see Supplementary Figs. 2 and 3. There was an improvement in prediction for all the models up to 100 observations, except for Logistic Regression and KNN. After that, a plateau was reached where AUROCs essentially stagnated between 0.8 and 0.9, regardless of the increase in the number of observations. Support Vector Classifier predictions were the most stable; see Supplementary Fig. 2.

For the AUCPR values, the plateau was reached sooner, around 75 observations, see Supplementary Fig. 3. Similarly, for the AUROC values, SVC predictions were the most stable and Logistic Regression fluctuated the most.

## Discussion

This study investigated the application of ML algorithms to predict the outcomes (success and failure) of the SBT based on a wide variety of biosignals, and irrespective of the cause of intubation. Preprocessing methodologies enabled us to include all types of data in the computations. We found that patients who did not pass the SBT spent a longer time on MV (measured as time between intubation and the first test). It is well-established that delaying weaning significantly increases the risk of complications associated with MV, such as amyotrophy, VAP and delirium [8, 43]. An algorithm could be used to predict the success of the weaning test in real time. This study suggests it may be important to look for the presence of VAP and significant weight gain before carrying out the SBT, as well as examining biosignals recorded in the 24 h before the test, to assess its likely success. The use of variables derived from routinely collected data could therefore assist in the management of critically ill patients. It could potentially reduce the duration of invasive ventilation and associated complications, for example, if ML models can predict SBT success more reliably than current methods. Furthermore, such algorithms would make a significant medical and economic contribution by reducing the length of hospital stays. Further studies will be needed to investigate these possibilities.

It is an original study in terms of its methodology. A few authors have investigated the application of ML models to weaning from MV, but without advanced AI methodologies (no handling of unbalanced datasets, no data cleaning, etc.) [17, 18]. Without such methodology, the use of biosignals in MV weaning is not well-studied in the literature [33, 44].

The AUROC and AUCPR of the SVC model were superior to those of the other ML algorithms (including the ensemble model). The use of imputation with KNN, oversampling with SMOTE and light dimensional reduction of time series data by FRESH appear to be effective techniques for obtaining good predictions.

Our results show that key variables contributing to the model predictions are the presence of VAP before the SBT, fibrinogenemia at the time of SBT, weight gain since admission and the respiratory rate in the 24 h prior to the SBT. The importance of respiratory rate seems obvious, as a high respiratory rate prior to the weaning test will induce patient exhaustion and increase the risk of failure. By highlighting these variables, we can influence the success of the weaning test. These are variables that are directly under the control of the clinician. For example, to achieve a favorable hydrosodic balance, it is possible to induce diuresis and reduce water intake. Preventing VAP, recognizing it early and treating it appropriately can have a direct effect on weaning success. It is also possible to act directly on respiratory rate, heart rate or blood pressure by initiating appropriate treatment, such as treating delirium, opioid withdrawal syndrome or hypertension for example.

Predicting the success of MV weaning using parameters that are routinely collected may enable better care. However, a prospective study with more data is needed.

### Strengths

A strength of this study is the inclusion of patients from two centers (Archet Hospital and Pasteur Hospital, Nice), which makes the results more generalizable.

The inclusion of patients admitted to ICU for different etiologies is also a strength of this study. By including patients ventilated for respiratory, neurological or for cardiopulmonary arrest reasons, this algorithm can be applied to any patient admitted to an ICU and requiring MV.

In comparison with other research in the field of MV weaning, we found a similar success/failure rate in our development cohort [4, 8, 17]. To reduce the bias that can result from such an unbalanced dataset (e.g., biased model and poor generalization), the SMOTE technique was used, which augments the minority class by creating artificial observations [39–41]. SMOTE has also proven effective in reducing overfitting (when the predicted model corresponds too closely to the training dataset and fails to generalize to new data) [45, 46].

We decided to use the AUCPR as the evaluation metric. The AUCPR is more suitable than AUROC for unbalanced data sets [47–49].

We have used simple variables that do not require additional measurements (such as blood tests, radiology, etc.) compared to what is done routinely. In fact, we have only integrated into our algorithms transformations of variables that were already available to us.

Trudzinski et al. note that determining the risk factors for weaning failure is complicated due to the number of studies and their heterogeneity. They conclude that multidimensional scores may be more useful in patient assessment [50]. Machine learning models provide a tool to analyze existing data in a systematic and consistent way and assist with its interpretation through multidimensional models.

The last and most important strength of this study is the rigorous framework development. Our data preprocessing using the imputation, FRESH and SMOTE techniques, enabled us to combine discrete variables and time series covering a broad range of patient characteristics. The resulting ML models predicted the success or failure of the SBT for patients with high accuracy. This methodology allowed us to obtain robust results that were superior to those of other studies looking at the use of ML in weaning, for example Lin et al. (AUROC: 0.908, 95% CI 0.864–0.943 for XGBOOST model) and Liu et al. (AUROC: 0.61, 95% CI 0.58–0.64 for Support Vector Machine model) [17, 18]. Compared to the recent study by Park et al., using ventilator data in a similar context with a multi-layer perceptron, our results are superior and more consistent (AUCPR 0.767, 95% CI 0.434–0.983) [34]. Our methodology allowed us to use weak signals (a signal that is difficult to “hear” and understand), signals that have not been widely used in medicine, because they are complex to use and understand [27, 51]. To avoid the phenomenon of the ML model learning from the SBT, we excluded data recorded after the test began. In this way we limited overfitting on our dataset [52].

### Limitations

The size of the patient sample included in this study is relatively small. This is due to the difficulty and time required to compile the various biosignals of interest for a sufficient number of patients. Patients admitted for very short stays with little or no respiratory failure (e.g., patients admitted for voluntary drug intoxication) did not require intubation or extensive monitoring. However, this sample size was sufficient to draw conclusions using ML models. A larger number (several thousand observations) would have been necessary to assess deep learning models. The number of patients was also consistent with the literature on similar study on different topics [24]. Furthermore, as shown in the supplementary analyses, beyond a hundred observations, the number of individuals appears to have little effect on the prediction accuracy.

The study was originally planned to use the data from around 500 patients. However, only 232 patients were included in the database. The main reason for this difference in sample size is that records prior to January 2020 had high levels of missing data. It was decided not to include records before this date to avoid impacting the training of the models and consequently the results obtained. To address the issue of the reduced sample size, an additional analysis was performed (see Supplementary Figs. 2 and 3) which showed that there was no significant improvement in results with an increased sample size beyond 100 patients.

The generation of a large number of explanatory variables can make the interpretation of the generated model difficult. This is why we opted to use the FRESH method to reduce the dimensionality of the temporal variables [53, 54].

The retrospective nature of the study can also be considered a limitation. However, this limitation is due to the design of the study, which specifically aimed at the development of the ML predictive algorithm. A second validation study will be performed using an external database to support the results of our analysis.

### Future work

A future prospective, multicenter study is planned that will evaluate the application of the ML algorithm in real time. In addition, future work will focus on determining the optimal time period for measuring the biosignals prior to the SBT (e.g., 48 h, 12 h, 2 h) to obtain the most accurate predictions of success. The implementation of these models into internal electronic systems will be the final goal. Using variables derived from commonly collected data should make this easier. In fact, the models only use measures that are already available and do not require any additional invasive intervention. We can envision a future implementation in our electronic clinical system or ventilator system for example. In the long term, the use of an optimized algorithm could potentially decrease LOS and MV durations by identifying the optimal timing for weaning from MV.

## Conclusion

This original study, in terms of its methodology and research topic, showed an application of different ML models to predict SBT success for ICU patients, regardless of etiology. We demonstrated that the combined use of discrete variables (e.g., VAP, weight gain, etc.) and continuous variables (biosignals), along with data preprocessing techniques (imputation by KNN, dimensional reduction of the temporal variables by FRESH, and oversampling by SMOTE), produced better predictions than previous results in the literature. Furthermore, this work enabled us to use existing data and highlighted the potential usefulness of weak signals in intensive care. However, further studies on large external databases will be necessary to validate these results.

## Supplementary Information

Supplementary Material 1.

Supplementary Material 2. Figure 1. The importance of different features in the models, expressed in SHAP values. We used light dimensional reduction. The highest SHAP values are at the top and the lowest at the bottom. Only the most important SHAP values are shown. The color code represents the feature value. The RR variables were features extracted from time series. RR: Respiratory Rate, c3: the c3 statistic measures non linearity in the time series, SHAP: SHapley Additive exPlanations, VAP: Ventilator-Associated Pneumonia.

Supplementary Material 3. Figure 2. Effect of varying sample size on AUROC for the different models in the test dataset. We used light dimensional reduction and the SMOTE technique. The total corresponds to N=232 observations. AUROC: Area Under the Receiver Operating Curve, KNN: K nearest neighbors, LGBM: Light Gradient Boosting Machine, SVC: Support Vector Classifier, XGBoost: Extreme Gradient Boosting.

Supplementary Material 4. Figure 3. Effect of varying sample size on AUCPR for the different models in the test dataset. We used light dimensional reduction and the SMOTE technique. The total corresponds to N=232 observations. AUCPR: Area Under Curve Precision-Recall, KNN: K nearest neighbors, LGBM: Light Gradient Boosting Machine, SVC: Support Vector Classifier, XGBoost: Extreme Gradient Boosting.

## Data Availability

The data that support the findings of this study are not openly available due to reasons of sensitivity and are available from the corresponding author upon reasonable request.

## Abbreviations

AI: Artificial intelligence
AUCPR: Area under the precision-recall curve
AUROC: Area under the receiver operating curve
BMI: Body mass index
COPD: Chronic obstructive pulmonary disease
CPIS: Clinical Pulmonary Infection Score
CRP: C-reactive protein
FRESH: FeatuRe Extraction based on Scalable Hypothesis tests
ICU: Intensive care unit
KNN: K-nearest neighbors
LGBM: Light gradient boosted machine
LOS: Length of stay
LR: Logistic regression
ML: Machine learning
MV: Mechanical ventilation
PCT: Procalcitonin
PEEP: Positive end expiratory pressure
PNV: Predictive negative value
PPV: Predictive positive value
RASS: Richmond Agitation Sedation Scale
RFC: Random Forest Classifier
RR: Respiratory rate
SAPSII: Simplified Acute Physiology Score II
SBT: Spontaneous breathing trial
SHAP: SHapley Additive exPlanations
SMOTE: Synthetic minority oversampling technique
SOFA: Sepsis-Related Organ Failure Assessment
SVC: Support vector classifier
VAP: Ventilator-associated pneumonia
VCV: Volume controlled ventilation
XGBoost: EXtreme gradient boosting
ZEEP: PEEP of zero
