# Supplementary material for: Using weak signals to predict spontaneous breathing trial success: a machine learning approach
Source: Intensive Care Med Exp. 2025 Mar 18;13:34. doi: 10.1186/s40635-025-00724-0 (PMC11920562; doi:10.1186/s40635-025-00724-0)
Supplement: Supplementary file 1 — Supplementary Material 1. [file 40635_2025_724_MOESM1_ESM.docx]

**Supplementary Methods :**

1. Oversampling method :

An unbalanced dataset, where one class (in this case, successful weaning from mechanical ventilation) is overrepresented, carries the risks of overfitting. To address this, two sampling techniques are commonly used: undersampling (reducing the majority class) and oversampling (increasing the minority class). Given the small sample size, oversampling was preferred in this ~~case~~ study. We used SMOTE (Synthetic Minority Oversampling Technique), which generates ~~virtual~~ synthetic individuals to enhance minority class representation.

SMOTE ~~works by synthetically generating~~ generate new minority class instances synthetically rather than simply duplicating existing ones, as random oversampling would do [1]. The algorithm generates these synthetic examples by interpolating between existing minority class ~~examples~~ instances. It selects a random sample from the minority class and then identifies its k-nearest neighbors (typically 5 by default). SMOTE then generates ~~creates~~ a synthetic instance by ~~choosing~~ selecting one of these neighbors and ~~generating~~ creating a new instance that lies along the line segment connecting the original instance and its neighbor.

The process can be broken down as follows:

- For a minority class instance $x$, SMOTE selects one of its nearest neighbors $x$_nn_ ​ from the same class.
- A new synthetic sample $x_{new}$ is generated according to the formula:

$$x_{new}=x+ \lambda\times(x_{nn}-x)$$

where $\lambda$ is a random number between 0 and 1. This ensures that the synthetic sample lies somewhere along the line segment between $x$ and $x$_nn_​, increasing diversity in the minority class without simply replicating existing instances.

1. Time series managements :

Some time series management tools (for biosignals) were used and implemented to complete this work. We applied ~~a~~ measurement interval homogenisation (based on the shortest measurement interval, *i.e.* a minute). For the series with ~~a~~ longer measurement intervals, ~~we repeated~~ the values were repeated to ~~correspond~~ match ~~to~~ the shortest measurement interval.

1. Feature extraction :

To ~~have~~ prepare time series data ~~that can be~~ for input into ML models, we ~~used~~ employed the Feature Extraction based on Scalable Hypothesis tests (FRESH) method [2–4]. The first step of the FRESH method is based on feature (variables) extraction. The extraction is the result of various calculations (like kurtosis, Fourier coefficients of the one-dimensional discrete Fourier transform, permutation entropy). This extraction generates several thousand features. To ~~decrease~~ reduce the training time of the ML algorithm, ~~a~~ dimensional reduction is applied. This is the second step: a variable or feature *X* is considered relevant to the prediction of *Y* (target), if and only if *X* and *Y* are not statistically independent. To this end, each variable is treated separately ~~by~~ using a univariate statistical test. To reduce inflation of the alpha risk, and the False Discovery Rate, ~~the~~ p-values are corrected using the Benjamini Yekutieli method. For this step, we only ~~took~~ selected features with a p-value < 0.05, it is the “light dimensional reduction”. We tested another dimensional reduction, limited to the first 20 variables (including both continuous and discrete variables), ~~the~~ known as “heavy dimensional reduction”.


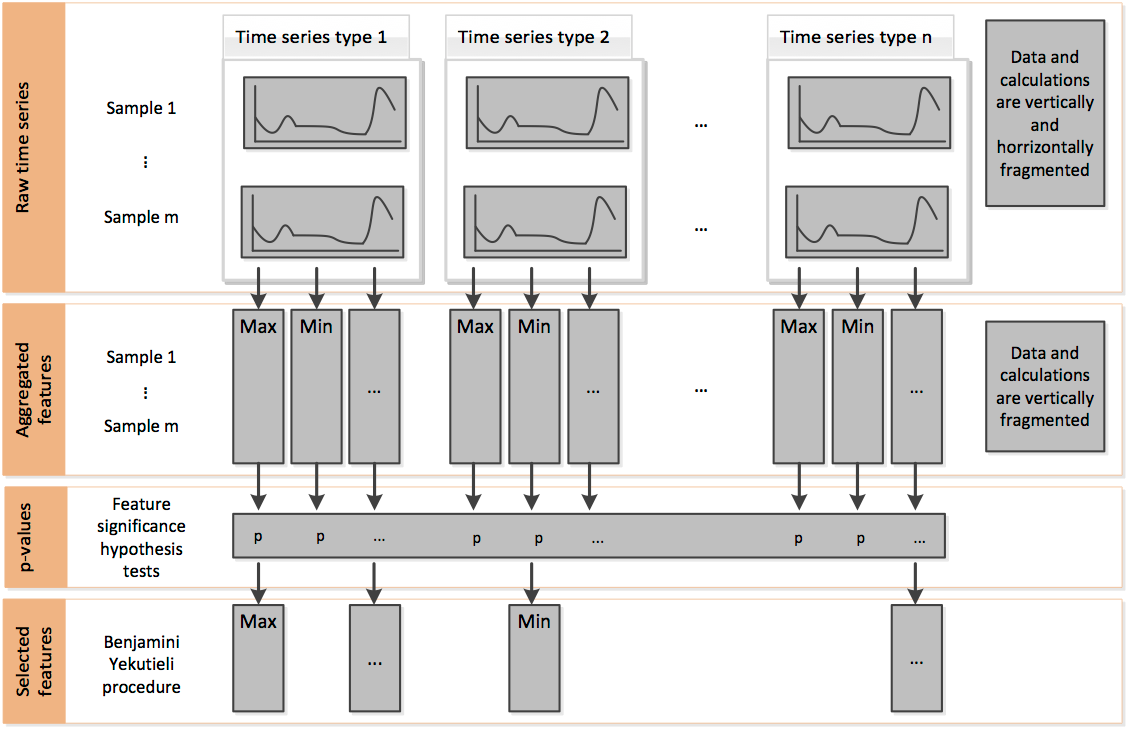


Supplementary method figure 1 : Data processing tiers of the filtered feature extraction algorithm.

Figure extracted from : Christ, M., Kempa-Liehr, A. W., and Feindt, M., “Distributed and parallel time series feature extraction for industrial big data applications » doi:10.48550/arXiv.1610.07717.

1. Shapley additive explanations

Shapley Additive Explanations (SHAP) provides a method for evaluating the importance of features in machine learning models [5, 6]. It decomposes model output into the contributions of each feature, assigning a value to each feature's effect on the output. This approach is applicable across various model types. For each instance, SHAP assigns an additive importance score (the SHAP value) to each feature, indicating its contribution to moving the model’s prediction away from the expected value (the average model prediction across the dataset).

The SHAP value decomposition is expressed as:

$$f\left( x \right)= \phi_{o}+\sum_{i=1}^{n} \phi_{i}$$

$f\left( x \right)$is the model’s prediction for instance $x$, $\phi_{o}$ is the baseline or expected value (the average prediction), $\phi_{i}$ is the SHAP value for feature $i$, representing its contribution to the prediction.

A high SHAP value indicates that the variable is very important in the model.

References :

1. Chawla NV, Bowyer KW, Hall LO, Kegelmeyer WP (2002) SMOTE: Synthetic Minority Over-sampling Technique. J Artif Intell Res 16:321–357. https://doi.org/10.1613/jair.953

2. Christ M, Braun N, Neuffer J, Kempa-Liehr AW (2018) Time Series FeatuRe Extraction on basis of Scalable Hypothesis tests (tsfresh – A Python package). Neurocomputing 307:72–77. https://doi.org/10.1016/j.neucom.2018.03.067

3. Christ M, Kempa-Liehr AW, Feindt M (2016) Distributed and parallel time series feature extraction for industrial big data applications. https://doi.org/10.48550/ARXIV.1610.07717

4. Christ M, Kempa-Liehr AW, Feindt M (2017) Distributed and parallel time series feature extraction for industrial big data applications

5. Shapley LS (1953) 17. A Value for n-Person Games. In: Kuhn HW, Tucker AW (eds) Contributions to the Theory of Games (AM-28), Volume II. Princeton University Press, pp 307–318

6. Lundberg S, Lee S-I (2017) A Unified Approach to Interpreting Model Predictions
