# Supplementary figures and images for: Using weak signals to predict spontaneous breathing trial success: a machine learning approach
Source: Intensive Care Med Exp. 2025 Mar 18;13:34. doi: 10.1186/s40635-025-00724-0 (PMC11920562; doi:10.1186/s40635-025-00724-0)

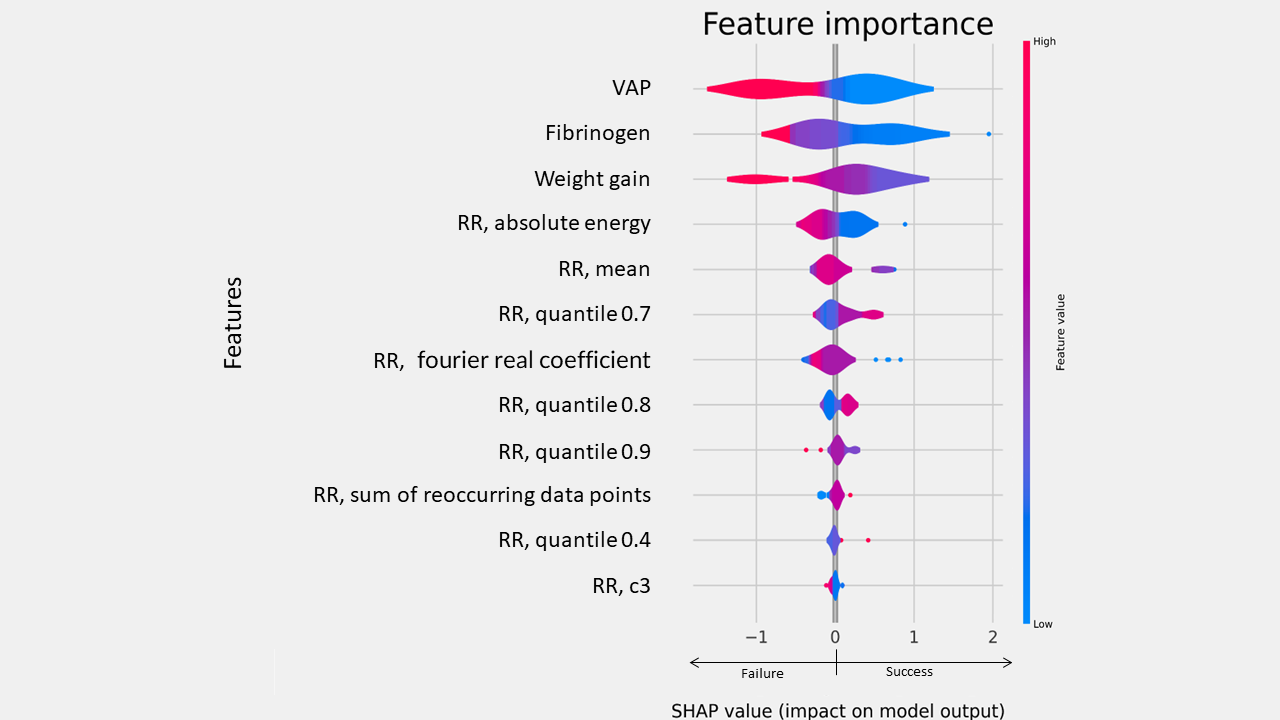

Supplement: Supplementary file 2 — Supplementary Material 2. Figure 1. The importance of different features in the models, expressed in SHAP values. We used light dimensional reduction. The highest SHAP values are at the top and the lowest at the bottom. Only the most important SHAP values are shown. The color code represents the feature value. The RR variables were features extracted from time series. RR: Respiratory Rate, c3: the c3 statistic measures non linearity in the time series, SHAP: SHapley Additive exPlanations, VAP: Ventilator-Associated Pneumonia. [file 40635_2025_724_MOESM2_ESM.tif]

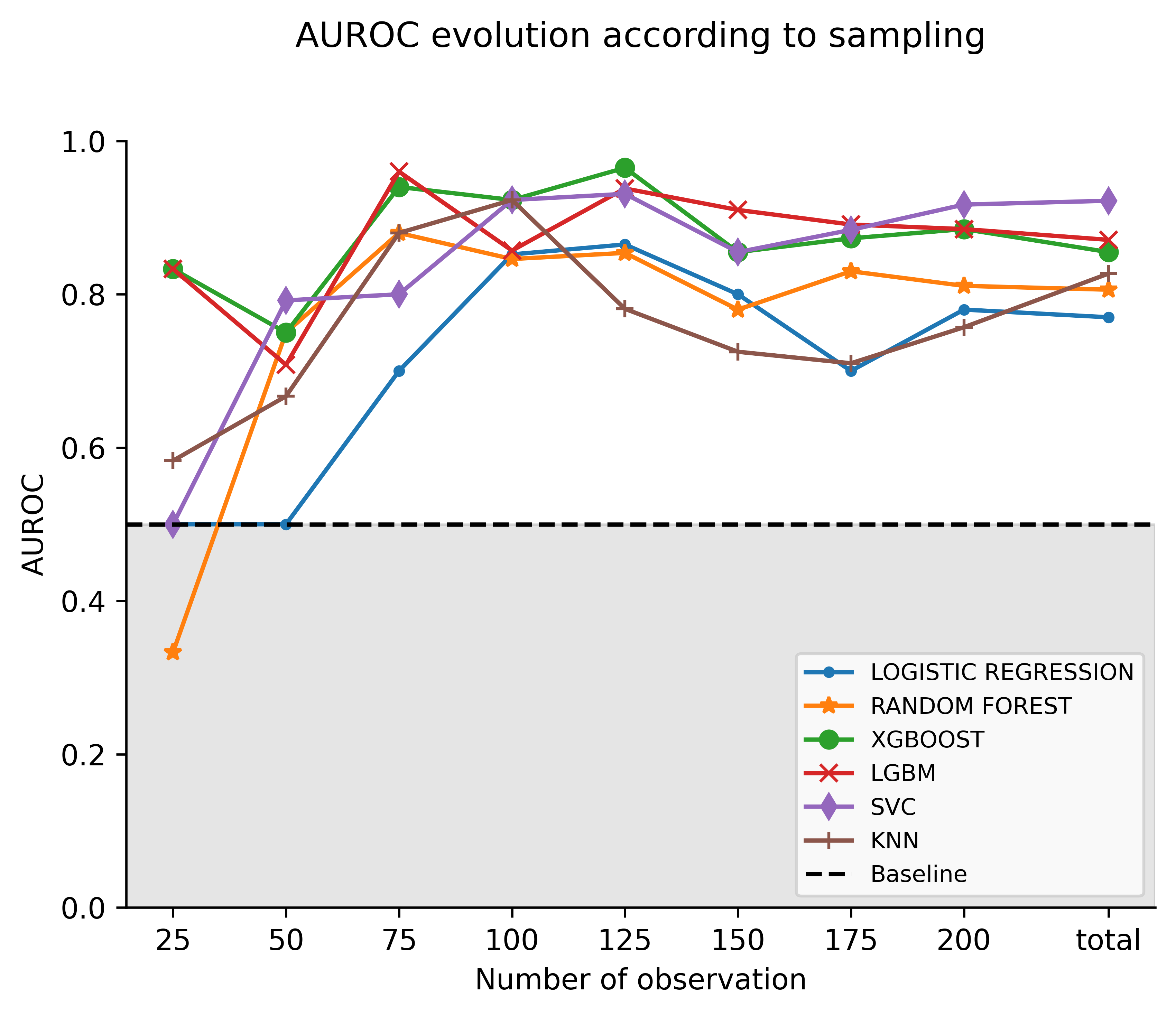

Supplement: Supplementary file 3 — Supplementary Material 3. Figure 2. Effect of varying sample size on AUROC for the different models in the test dataset. We used light dimensional reduction and the SMOTE technique. The total corresponds to N=232 observations. AUROC: Area Under the Receiver Operating Curve, KNN: K nearest neighbors, LGBM: Light Gradient Boosting Machine, SVC: Support Vector Classifier, XGBoost: Extreme Gradient Boosting. [file 40635_2025_724_MOESM3_ESM.tif]

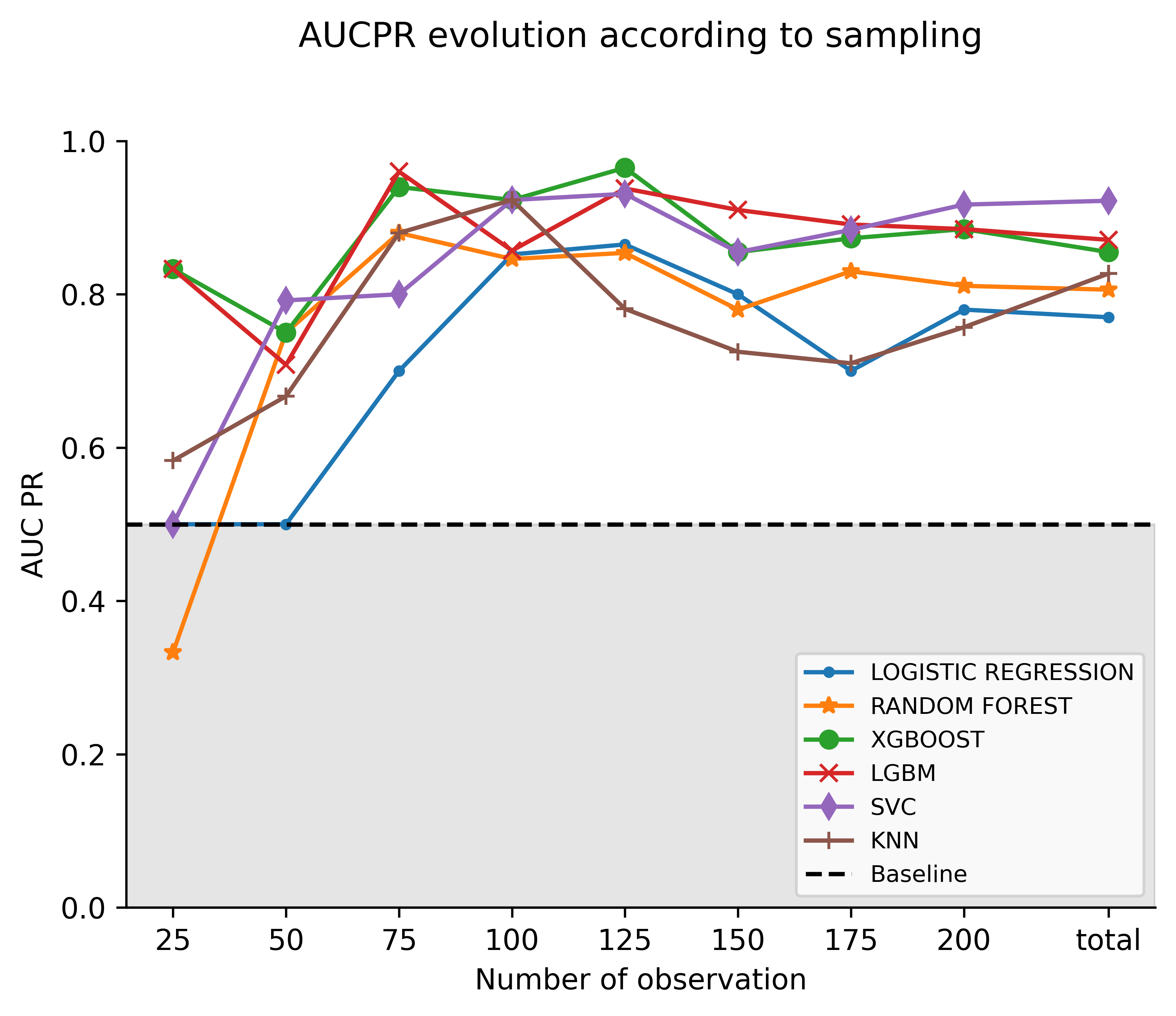

Supplement: Supplementary file 4 — Supplementary Material 4. Figure 3. Effect of varying sample size on AUCPR for the different models in the test dataset. We used light dimensional reduction and the SMOTE technique. The total corresponds to N=232 observations. AUCPR: Area Under Curve Precision-Recall, KNN: K nearest neighbors, LGBM: Light Gradient Boosting Machine, SVC: Support Vector Classifier, XGBoost: Extreme Gradient Boosting. [file 40635_2025_724_MOESM4_ESM.tif]
